# Psychometric properties of comprehensive cognitive, affective, and psychomotor competency assessment scales in psychodynamic psychotherapy for borderline personality disorder

**DOI:** 10.3389/fpsyt.2024.1389992

**Published:** 2024-12-06

**Authors:** Petrin Redayani Lukman, Tjhin Wiguna, Diantha Soemantri, Sri Linuwih Menaldi, Sylvia Detri Elvira, Limas Sutanto, Tuti Wahmurti A. Sapiie, Aria Kekalih, Reina Rahma Noviasari, Hukma Shabiyya Rizki, Kharisma Zatalini Giyani

**Affiliations:** ^1^ Doctoral Program in Medical Sciences, Faculty of Medicine, Universitas Indonesia, Jakarta, Indonesia; ^2^ Department of Psychiatry, Faculty of Medicine, Universitas Indonesia, Dr. Cipto Mangunkusumo General Hospital, Jakarta, Indonesia; ^3^ Department of Medical Education, Faculty of Medicine, Universitas Indonesia, Jakarta, Indonesia; ^4^ Department of Dermatology and Venereology, Faculty of Medicine, Universitas Indonesia, Dr. Cipto Mangunkusumo General Hospital, Jakarta, Indonesia; ^5^ Division of Psychoanalysis in Psychiatry, Psychotherapy Section, Indonesian Psychiatric Association, Jakarta, Indonesia; ^6^ Department of Psychiatry, Faculty of Medicine, University of Padjajaran, Bandung, Indonesia; ^7^ Department of Community Medicine, Faculty of Medicine, Universitas Indonesia, Jakarta, Indonesia

**Keywords:** psychodynamic psychotherapy, borderline personality disorder, assessment scale, validity, psychometric

## Abstract

**Background:**

Psychodynamic psychotherapy is a type of psychotherapy for individuals with borderline personality disorder (BPD). However, competency in conducting effective psychodynamic psychotherapy for BPD is difficult to evaluate. Therefore, this study aimed to identify the psychometric properties of a comprehensive scale to assess cognitive, affective, and psychomotor competencies (CS-CAPC) in psychodynamic psychotherapy for BPD.

**Methods:**

This is a qualitative study. The first step used the Delphi technique to gather experts’ opinions on the cognitive, affective, and psychomotor competencies necessary to conduct psychodynamic psychotherapy for BPD. The experts comprised three psychotherapists, seven psychiatrists with experience in psychotherapy, and nine teaching staff. A panel discussion was conducted to obtain qualitative data. Thematic data analysis was adopted, and content validity testing was used to analyze the content validity of the CS-CAPC in psychodynamic psychotherapy for BPD.

**Results:**

The CS-CAPC comprised two scales assessing two specific competencies in psychodynamic psychotherapy for BPD: The first scale, the psychodynamic formulation competency assessment scale (PF-CAS), comprised six items, including the case description, etiology, and potential course of therapy. The second scale, the practical-competency assessment scale (PC-CAS) for psychodynamic psychotherapy for BPD, comprised 12 items, including building a therapeutic alliance, performing psychodynamic interventions while working through the therapeutic process, and closing the session. The scale-level content validity index (S-CVI) for the PF-CAS was 0.981, and that for the PC-CAS in psychodynamic psychotherapy for BPD was 1.00.

**Conclusion:**

The CS-CAPC in psychodynamic psychotherapy for BPD had good validity in assessing individual competency in the cognitive, affective, and psychomotor domains.

## 1 Introduction

Borderline personality disorder (BPD) is difficult to treat. Patients with BPD have complex symptoms that can make them uncooperative, extremely sensitive, and exhibit alternating positive and negative emotions that often inflict intense negative emotional reactions on therapists, such as frustration, anger, or feeling incompetent (1). Prevalence studies suggest that the prevalence of BPD in the general population is approximately 1.6% to 1.8%. The prevalence of this disorder is higher in psychiatric outpatient populations (approximately 11%) and reaches 20% in psychiatric inpatient population (2–4). In Indonesia, although no nationwide data are available on the prevalence of BPD, there appears to be an increase in the number of patients with BPD, according to data from the psychiatric clinic and ward of Indonesia’s national referral center, Dr. Cipto Mangunkusumo National Central General Hospital (RSUPN-CM), Jakarta, Indonesia. In 2020, 242 clinic visits were recorded from 71 patients, including four new patients. Additionally, 25 cases of hospitalization of patients with BPD were recorded. In 2021, the number of polyclinic visits and hospitalizations increased to 775 clinic visits and 190 patients, including 26 new patients in polyclinics and 68 inpatient hospitalization cases (5).

Psychotherapy and psychopharmacology are both used to assist individuals with BPD (6). Psychodynamic psychotherapy can be applied as a psychotherapeutic approach, whose main goals in individuals with BPD include improving self-cohesion, self-other representations, affect regulation, and mentalization capacity by helping them attain a better understanding of their own and others’ experiences (7). Moreover, it is effective in reducing suicidal behavior, anger, and impulsivity, as well as in enhancing psychosocial functioning (8). However, several challenges may also be encountered during psychodynamic psychotherapy, such as intense transference and countertransference, which can hinder the therapist-patient relationship. Therefore, understanding and managing the transference-countertransference dynamics in therapy present central strategies in conducting psychodynamic psychotherapy for BPD to prevent such reactions from disrupting the therapeutic relationship and subsequently improve therapeutic benefits. Therefore, to be psychodynamic psychotherapists for individuals with BPD, therapists require self-awareness, perseverance, and neutrality to survive intense transference-countertransference reactions and strike a sound balance between empathy and detachedness (9, 10).

The Indonesian College of Psychiatry has defined the competency of performing psychodynamic psychotherapy as a general competency that must be achieved by psychiatry residents who become full-fledged therapists once they graduate and can perform psychodynamic psychotherapy for BPD (11). Therapist competence has been conceptualized as the level of the therapist’s knowledge and skill to implement a treatment to an acceptable standard (12, 13). The need to evaluate therapists’ competence in psychotherapy has been widely recognized (14). Psychodynamic psychotherapists’ competencies are essential as higher levels of competence lead to better patient outcomes, such as clinical symptoms and social functioning (15, 16). However, deficiencies or misapplication of these techniques are related to negative therapy outcomes (17). Assessment of psychotherapeutic competence could be achieved through knowledge tests, evaluation of patient outcomes, patient feedback, or evaluation of treatment sessions using evaluation scales (12, 15, 18). Structured evaluation scales facilitate a systematic assessment of competence (19). Using a competency assessment scale also enables the provision of performance-based feedback for trainee psychiatrists (20). Most of these scales are constructed as a rating scale to be used by supervisors based on their observation of the psychotherapists’ performance and are developed based on expert consensus or the authors’ expertise (13, 21). Although several standardized psychotherapy competency assessment scales have been developed for psychotherapists, these are primarily used in general practice, such as the Cognitive Therapy Scale-Revised or the Supervisor’s Evaluation Scale (15, 22–24). Several competency assessment scales have been developed to assess psychotherapeutic competency based on distinct mental disorders. Currently, standardized competency assessment scales for evaluating psychotherapists’ competency in conducting psychodynamic psychotherapy for BPD are limited.

Moreover, limited psychotherapy competence scales exist that implement the assessment of the three domains of learning, namely the cognitive, affective, and psychomotor domains, as defined by Bloom in the Taxonomy of Educational Objectives. Each learning domain comprises hierarchical categories of learning objectives ranging from the simplest to the most complex, which can be applied to assessment scales (25). The application of Bloom’s domains of learning in medical education has helped establish a model of clinical competence as a combination of intellectual knowledge, attitudes or awareness of values, and psychomotor abilities (26). Assessments should involve all domains of learning and specify the learning objectives that must be achieved so that the competence being assessed can be comprehensively measured (27). Additionally, a method of assessment should possess good psychometric properties, such as validity and reliability, to ensure its ability to correctly measure the intended performance, and the results should be consistent across different instances and raters (15, 28). Therefore, it is essential to develop comprehensive scales to assess cognitive, affective, and psychomotor competency (CS-CAPC) in psychodynamic psychotherapy for BPD to determine the competency of psychodynamic psychotherapists who conduct psychodynamic psychotherapy in individuals with BPD. The CS-CAPC comprises two scales. The first scale, the psychodynamic formulation competency assessment scale (PF-CAS), was developed to assess the skill of creating a psychodynamic formulation by evaluating the therapist’s written psychodynamic formulation. The second scale, the practical competency assessment scale (PC-CAS) in psychodynamic psychotherapy for BPD, was developed to assess therapists’ skills in performing psychodynamic psychotherapy for patients with BPD in real-life clinical settings by assessing a video recording of a psychotherapy session or by direct observation of a session. The PF-CAS was designed for cognitive and affective domain evaluations, whereas the PC-CAS in psychodynamic psychotherapy for BPD was designed to assess the cognitive, affective, and psychomotor domains.

## 2 Materials and method

### 2.1 Study design

The development of the CS-CAPC comprises two phases: (1) data collection and (2) measurement of the validity of the scales. For data collection, an initial item list was developed based on a literature review and Delphi survey, and a panel discussion was held to explore experts’ and stakeholders’ perceptions of the required characteristics and components of the scales. The validity of the instruments was tested using content validity testing. This study was conducted between September 2022 and August 2023 using Zoom’s online discussion platform and distributing online forms via email.

### 2.2 Ethics statement

Ethical approval was obtained from the Health Research Ethics Committee of the Faculty of Medicine, Universitas Indonesia (protocol number: 22-10-1201; KET-1074 UN2.F1/ETIK/PPM.00.02/2022).

### 2.3 Participants and procedure

Psychotherapy experts and stakeholders in psychiatric education were invited to provide their perceptions and feedback on these scales. We utilized criterion-based purposive sampling to recruit participants based on their experience and expertise in relevant fields. Each part of the study involved a different set of participants. All participants were invited to participate in the study and were informed of its aims and procedures. Written informed consent was obtained from all participants before their enrolment in the study.

#### 2.3.1 Delphi survey

The Delphi technique is a common method used to achieve consensus among a panel of experts or stakeholder groups. It is performed by conducting iterative rounds of surveys until a consensus is reached (29). Seven psychiatrists from faculty members of psychiatry residency-program institutions in Indonesia and council members of the Psychotherapy Section of the Indonesian Psychiatric Association were invited to participate in the survey.

For the Delphi survey, we developed the initial item lists for the PF-CAS and PC-CAS in psychodynamic psychotherapy for BPD based on a literature review. These scales were designed and intended for use in the Indonesian language. Each item on the list was accompanied by a short description of the level of cognitive, affective, or psychomotor learning to be achieved. There were seven items in the PF-CAS and 12 items in the PC-CAS in psychodynamic psychotherapy for BPD. An online Delphi survey was conducted by sending initial item lists to seven experts via email. In each round, the experts rated the items on a 4-point Likert scale of 1 (strongly disagree), 2 (disagree), 3 (agree), and 4 (strongly agree). Moreover, the experts were free to provide written suggestions, such as revisions, deletions, or additions to the items. The principal investigators summarized the scores and feedback and returned the results to the experts in the next round until a consensus was reached. Descriptive statistics were used to calculate the experts’ mean scores. A consensus was defined as a mean score of ≥ 3.5 (30).

#### 2.3.2 Panel discussion

Following the Delphi method, finalized item drafts were developed into two scales in the form of evaluation rubrics. We designed the rubric components defined by Burghart and Panettieri (31): task description, scale of achievement, dimensions, and description of dimensions. The names of the scales represent task descriptions, whereas the evaluation items represent the dimensions of the task. Each item was quantitatively scored on a scale from 0 to 3, with 0 indicating the lowest and 3 indicating the highest score. Each score on the scale has a description. All the item scores were totaled and converted into numeric scores of 0–100 to determine the final score.

The panel discussion was conducted as a focus group discussion via the online discussion platform, Zoom Meetings, to reach an agreement on all scale items and descriptions. The panel discussion included three experts in psychotherapy from members of the Psychotherapy Section of the Indonesian Psychiatric Association. Inclusion criteria were (1) a consultant in psychotherapy, and (2) a faculty member of a psychiatric education institution or having experience in providing psychotherapy education through workshops. Exclusion criteria were (1) refusing to participate in the study, and (2) are no longer active as a clinician. There were one male expert and two female experts. The mean age was 56.7 years old. The experts had experience in psychotherapy ranging from 10 to 31 years, with a mean of 23 years. Data from the discussions were analyzed using thematic analysis. The resulting scales were then tested for content validity.

#### 2.3.3 Content validity testing

A content validity test for the CS-CAPC in psychodynamic psychotherapy for BPD was conducted to investigate each item’s relevance. We distributed the scales along with a content validation questionnaire via email to the participants, who then rated each item’s significance on a 4-point Likert scale: 1 (very irrelevant/VI); 2 (not relevant/NR); 3 (relevant/R); and 4 (very relevant/VR). Participants could also provide written feedback on the items.

Content validity was determined by calculating the item-level content validity index (I-CVI) for each scale item and the scale’s average scale-level (S-CVI/Ave) values. The I-CVI was calculated by dividing the number of participants who allocated a rating of 3 or 4 by the total number of participants. The S-CVI/Ave was calculated by averaging the I-CVI for all scale items. An I-CVI value of > 0.79 and an S-CVI/Ave value of > 0.78 were deemed acceptable (32).

Nine faculty psychiatrists who teach psychotherapy in their respective residency programs were invited to participate in the content validity testing. We employed stratified purposive sampling to recruit one psychotherapy teaching staff member from each of the nine psychiatric residency-program institution centers in Indonesia.

Two males and seven females were selected. Regarding age (years), one faculty psychiatrist was between 30 and 40, five were between 40 and 50, one was between 50 and 60, and two were between 60 and 70. Two were consultants in child and adolescent psychiatry, two were consultants in consultation-liaison psychiatry, and five were general psychiatrists. All had experience in teaching psychotherapy, with years of teaching experience ranging from 2 to 23 years and a mean of 14.5 years.

## 3 Results

### 3.1 Delphi survey

**Figure 1:**
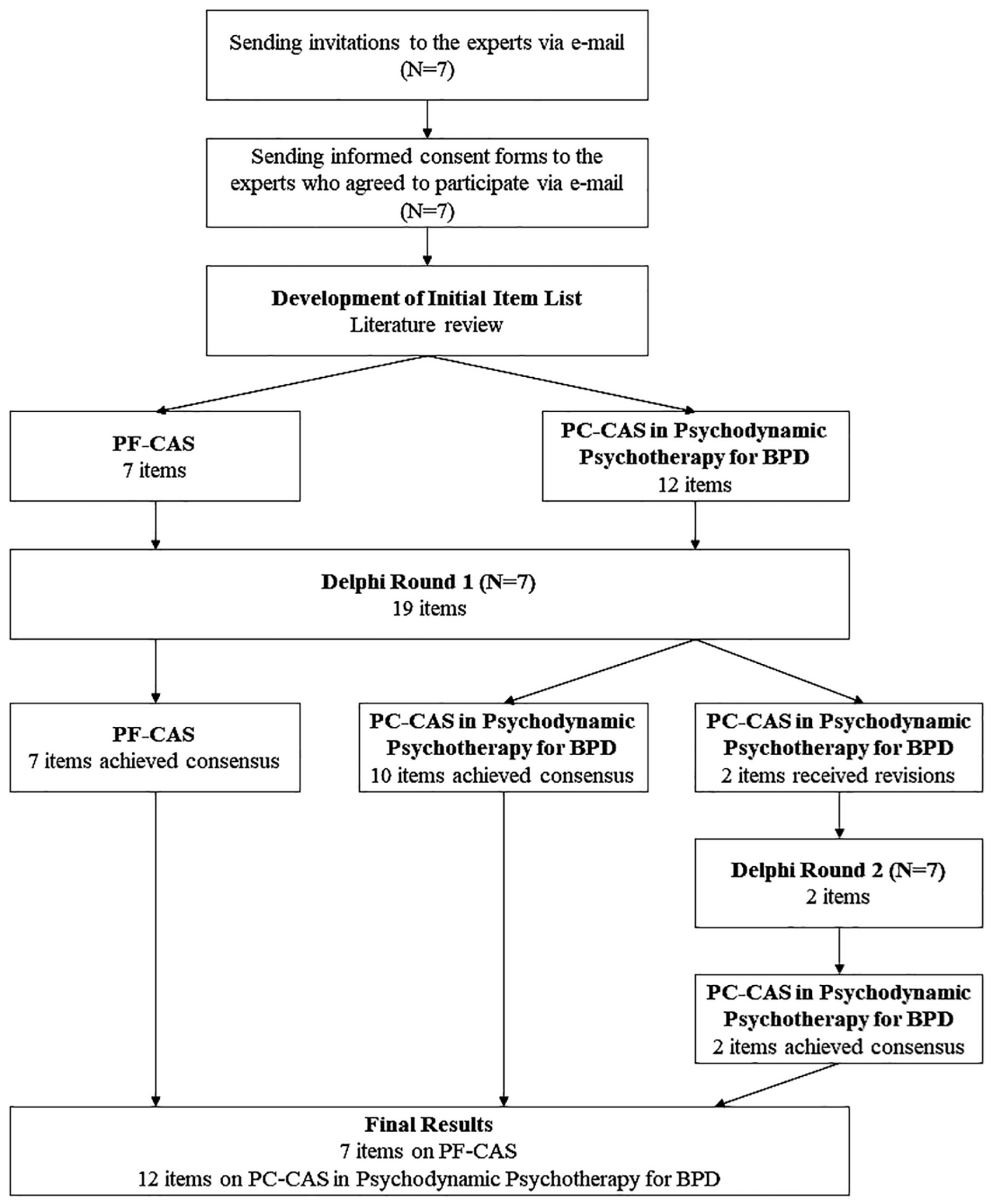
Delphi survey flowchart.

**Figure 1** illustrates the flow of the Delphi survey. Seven experts participated in the Delphi survey, with a mean age of 59.5 years and experience ranging from 5 to 31 years. Demographic characteristics of the experts are shown in **Table 1**. We conducted two rounds of the Delphi analysis. In the first round, all the items in PF-CAS achieved the consensus criteria of a mean score ≥3.5 and were accepted. For the PC-CAS in psychodynamic psychotherapy for BPD, one item (Item 2), “Showing a warm attitude to the patient,” obtained a mean score of 3.28, lower than the predefined criteria of consensus. The item was revised according to the written feedback and included in the second Delphi round: “Paying attention to the patient without judgment and prejudice.” The remaining items on PC-CAS in psychodynamic psychotherapy for BPD reached consensus with a mean score in the range 3.57–3.85. However, item 4, “Maintaining good eye contact throughout the therapy session (for at least 85% of the session),” received revision suggestions from two of the experts. Thus, we included the item in the second Delphi round. Therefore, the second Delphi round was held for the PC-CAS in psychodynamic psychotherapy for BPD items which still needed revisions.

**Table 1:** Demographic characteristics of Delphi survey participants (N=7).

Characteristics	M (SD)	n (%)
Age	59.5 (13.3)	
Gender		
Male		2 (29)
Female		5 (71)
Professional background		
General psychiatrist		1 (14)
Consultant in psychotherapy		4 (58)
Consultant in child and adolescent psychiatry		1 (14)
Consultant in biological psychiatry		1 (14)
Years of experience in psychotherapy	16.7 (11.5)	
5 to 15 years		4 (58)
16 to 25 years		1 (14)
25 years and above		2 (28)

The new version of the PC-CAS in psychodynamic psychotherapy for BPD, comprising the revised items, was sent back to the experts for the second Delphi round. A consensus was reached on all items, with a mean score of 3.71. Written feedback in the second round was used to develop descriptions of the item-scoring criteria for the study’s next step. **Table 2** shows the results of Delphi rounds 1 and 2. The resulting draft of the scales comprised 7 items on the PF-CAS and 12 items on the PC-CAS in psychodynamic psychotherapy for BPD (**Table 3**).

**Table 2:** Delphi Round 1 and 2 Results.

	Round 1 (N=7)					Round 2 (N=7)				
Items	Mean	% rated 3-4	Consensus	Revision Suggestion	Conclusion	Mean	% rated 3-4	Consensus	Revision Suggestion	Conclusion
PF-CAS										
1. Describing the patient’s condition and stressors	3.86	100	Yes	No	Accepted					
2. Formulating the biological, intrapsychic, and sociocultural factors that influence the patient’s condition	3.86	100	Yes	No	Accepted					
3. Determining the suitable psychoanalytic theory to explain the patient’s condition	3.71	100	Yes	Minor	Accepted					
4. Describing the potential resistance, transference, and countertransference that might occur in the therapy sessions	3.86	100	Yes	No	Accepted					
5. Explaining the management plan for the potential resistance, transference, and countertransference that might occur in the therapy sessions	3.86	100	Yes	No	Accepted					
6. Explaining the plan and goals of the psychotherapy	3.86	100	Yes	No	Accepted					
7. Explaining the patient’s prognosis in the course of the psychotherapy	3.71	100	Yes	No	Accepted					
PC-CAS in psychodynamic psychotherapy for BPD										
1. Beginning the session	3.86	100	Yes	No	Accepted					
2. Showing a warm attitude to the patient	**3.29**	85	**No**	**Major**	Revision needed					
**Revision for Round 2**: Paying attention to the patient without judgment and prejudice						3.71	100	Yes	No	Accepted
3. Conducting conversations that aim to build rapport with the patient	3.86	100	Yes	No	Accepted					
4. Maintaining good eye contact throughout the therapy session (for at least 85% of the session)	3.71	100	Yes	**Major**	Revision needed					
**Revision for Round 2**: Maintaining good eye contact throughout the therapy session for most of the time						3.71	100	Yes	No	Accepted
5. Aligning the gestures and posture per the patient’s state during the therapy session	3.86	100	Yes	No	Accepted					
6. Paraphrasing the patient’s statements to show that the therapist is empathetic and understanding toward the patient’s feelings	3.71	100	Yes	No	Accepted					
7. Using appropriate psychodynamic psychotherapy interventions per the patient’s state	3.86	100	Yes	No	Accepted					
8. Managing the patient’s resistance in the session	3.57	100	Yes	No	Accepted					
9. Responding adequately to the changes in the patient’s emotional states	3.86	100	Yes	No	Accepted					
10. Managing the patient’s transference	3.86	100	Yes	No	Accepted					
11. Managing countertransference reactions	3.86	100	Yes	No	Accepted					
12. Summarizing and closing the session	3.86	100	Yes	No	Accepted					

*Bold=The item did not reach the consensus of ≥ 3.5 or received major revision suggestions.

**Table 3:** The resulting draft of the scales from the Delphi survey.

Scale	Items
PF-CAS	1. Describing the patient’s condition and stressors 2. Formulating the biological, intrapsychic, and sociocultural factors that influence the patient’s condition 3. Determining the suitable psychoanalytic theory to explain the patient’s condition 4. Describing the potential resistance, transference, and countertransference that might occur in the therapy sessions 5. Explaining the management plan for the potential resistance, transference, and countertransference that might occur in the therapy sessions 6. Explaining the plan and goals of the psychotherapy 7. Explaining the patient’s prognosis in the course of the psychotherapy
PC-CAS in psychodynamic psychotherapy for BPD	1. Beginning the session 2. Paying attention to the patient without judgment and prejudice 3. Conducting conversations that aim to build rapport with the patient 4. Maintaining good eye contact throughout the therapy session for most of the time 5. Aligning the gestures and posture per the patient’s state during the therapy session 6. Paraphrasing the patient’s statements to show that the therapist is empathetic and understanding toward the patient’s feelings 7. Using appropriate psychodynamic psychotherapy interventions per the patient’s state 8. Managing the patient’s resistance in the session 9. Responding adequately to the changes in the patient’s emotional states 10. Managing the patient’s transference 11. Managing countertransference reactions 12. Summarizing and closing the session

### 3.2 Panel discussion

The items agreed upon in the Delphi survey were developed into two scales. Each item was scored on a scale of 0–3, with descriptions for each score and a description of the level of cognitive, affective, and psychomotor learning assigned to each item. The draft scales were then subjected to a panel discussion among the three experts.

Based on the panel discussion, the number of items in the new iteration of the PF-CAS changed from seven to six. Changes were made to Items 5 and 6, which were combined into one item. The changes were as follows: “Explaining the management plan for the potential resistance, transference, and countertransference that might occur in the therapy sessions” and “Explaining the plan and goals of the psychotherapy” were combined into “Explaining the goals of the psychotherapy and the management plan for the potential resistance, transference, and countertransference that might occur in the therapy sessions.” Accordingly, the description of Score 3 criteria in Item 5 was changed from “Clearly describes the management plan for the potential resistance, transference, and countertransference that might occur in the therapy sessions” to “Clearly describes the goals of the psychotherapy and the management plan for the potential resistance, transference, and countertransference that might occur in the therapy sessions.”

There are 12 items on the PC-CAS in psychodynamic psychotherapy for BPD. The panel discussion resulted in several revisions to the draft, namely, those to Items 6, 8, 10, and 11. The change to Item 6 was in the description of Score 1, from “The therapist rarely paraphrases” to “The therapist very rarely paraphrases or paraphrases too often.” For Item 8, a minor grammatical change was made to the description of Score 1 to increase the clarity of the sentence. In Item 10, the change in Score 3 was from “The therapist interprets and discusses the patient’s transference so that the patient understands themselves and their problems better” to “The therapist interprets and discusses the patient’s transference so that the patient gradually understands themselves and their problems better.” The final change in the scale was on Item 11 for the description of Score 1, from “The therapist appears to try managing their feelings and responds to the patient, but the response is inappropriate, and they seem to be unable to navigate the session,” to “The therapist appears to recognize their feelings and try to manage them, responds to the patient but the response is inappropriate, and they seem to be unable to navigate the session.”

### 3.3 Content validity testing

Both scales are deemed valid based on an S-CVI/Ave value of 0.981 for the PF-CAS and an S-CVI/Ave value of 1.00 for the PC-CAS in psychodynamic psychotherapy for BPD. The I-CVI values for all six items in the PF-CAS ranged from 0.889 to 1.00. All items received a score of 3 or 4, except for Item 5, which received eight scores of 3 or 4 and one of 2. Items 1, 4, and 6 received minor revisions from the participants. The scoring scale on Item 1 was modified for better specificity of the scale. Items 4 and 6 were revised for better wording.

The I-CVI for all twelve items in the PC-CAS was 1.00. All items attained a score of 3 or 4. Modifications were made to the scoring scale descriptions of Item 12 for better specificity of the scoring scale based on feedback from the three participants. **Table 4** shows the results of the content validity testing. Examples of the feedback and revisions can be seen in **Table 5**.

**Table 4:** Content validity testing results.

Items	Rater Scores									% rated 3-4	I-CVI	S-CVI/Ave
	R*1	R*2	R*3	R*4	R*5	R*6	R*7	R*8	R*9			
PF-CAS												
Item 1	3	3	3	3	4	3	4	4	4	100	1.00	0.98
Item 2	3	3	4	3	4	4	4	3	4	100	1.00	
Item 3	3	4	4	3	4	4	4	4	4	100	1.00	
Item 4	3	3	3	3	4	4	4	4	4	100	1.00	
Item 5	3	3	2	3	4	4	4	4	4	88	0.889	
Item 6	3	3	4	3	4	4	4	4	4	100	1.00	
PC-CAS in psychodynamic psychotherapy for BPD												
Item 1	3	4	4	3	4	4	4	3	4	100	1.00	1.00
Item 2	4	3	4	3	4	4	4	4	4	100	1.00	
Item 3	3	3	4	3	4	4	4	4	4	100	1.00	
Item 4	4	3	4	3	4	4	4	4	4	100	1.00	
Item 5	4	3	4	3	4	4	4	4	4	100	1.00	
Item 6	4	3	4	3	4	4	4	4	4	100	1.00	
Item 7	4	3	4	3	4	4	4	3	4	100	1.00	
Item 8	4	3	4	3	4	4	4	4	4	100	1.00	
Item 9	4	3	4	3	4	4	4	3	4	100	1.00	
Item 10	3	3	4	3	4	4	4	4	4	100	1.00	
Item 11	4	3	4	3	4	4	4	4	4	100	1.00	
Item 12	3	3	4	3	4	4	4	4	4	100	1.00	

*R, Rater.

**Table 5:** Examples of participants’ content validity feedback for the scales.

Original Items	Feedback	Final Version of the Item
*PF-CAS* Item 1, Score 1: The patient’s condition and stressors are described unclearly and not systematically; thus, they are not easy to understand.	“It is better to make the five points mentioned in Score 3 the reference points that need to be achieved, for a more systematic scoring scale. For example, Score 1: Describes 1 point out of 5 Score 2: Describes 2-3 points out of 5, and so on…”	*PF-CAS* Item 1, Score 1: The patient’s condition and stressors are described unclearly and not systematically; thus, they are not easy to understand. Only includes 1 out of 5 from the following: • The patient’s condition upon seeking help • The patient’s psychosocial condition • Overview of the patient’s developmental period • The patient’s support system • The stressors that exist within the patient
*PF-CAS* Item 1, Score 3: The patient’s condition and stressors are described clearly, systematically, and easily understood. Includes: • The patient’s condition upon seeking help • The patient’s psychosocial condition • Overview of the patient’s developmental period • The patient’s support system • The stressors that exist within the patient		*PF-CAS* Item 1, Score 3: The patient’s condition and stressors are described clearly, systematically, and easily understood. Includes 4–5 out of 5 from the following: • The patient’s condition upon seeking help • The patient’s psychosocial condition • Overview of the patient’s developmental period • The patient’s support system • The stressors that exist within the patient

The scales were revised and finalized, the finalized scales are shown in **Tables 6** and **7**.

**Table 6:** Psychodynamic formulation competency assessment scale.

No.	Evaluation Item	Score (0-3)	Level of Learning*
1	Describing the patient’s condition and stressors		C4: Analyzes the information
2	Formulating the biological, intrapsychic, and sociocultural factors that influence the patient’s condition		C4: Analyzes the information A2: Responds to the learning situation
3	Determining the suitable psychoanalytic theory to explain the patient’s condition		C3: Applies the information A2: Responds to the learning situation
4	Describing the potential resistance, transference, and countertransference that might occur in the therapy sessions		C6: Creates new knowledge A3: Values the learning
5	Explaining the goals of the psychotherapy and management plan for the potential resistance, transference, and countertransference that might occur in the therapy sessions		C6: Creates new knowledge A3: Values the learning
6	Explaining the patient’s prognosis in the course of the psychotherapy		C4: Analyzes the information
Total Score			

*Bloom’s Taxonomy: C, cognitive; A, affective; P, psychomotor.

**Table 7:** Practical competency assessment scale in psychodynamic psychotherapy for BPD.

No.	Evaluation Item	Score (0-3)	Level of Learning*
1	Beginning the session		C3: Applies the information P4: Mechanism (habitual use of the skill)
2	Paying attention to the patient without judgment and prejudice		C3: Applies the information A2: Responds to the learning situation P4: Mechanism (habitual use of the skill)
3	Conducting conversations that aim to build rapport with the patient		C4: Analyzes the information A2: Responds to the learning situation P4: Mechanism (habitual use of the skill)
4	Maintaining good eye contact throughout the therapy session for most of the time		C3: Applies the information A2: Responds to the learning situation P4: Mechanism (habitual use of the skill)
5	Aligning the gestures and posture per the patient’s state during the therapy sessions		C3: Applies the information A2: Responds to the learning situation P4: Mechanism (habitual use of the skill)
6	Paraphrasing the patient’s statements to show that the therapist is empathetic and understanding toward the patient’s feelings		C3: Applies the information A3: Values the learning P4: Mechanism (habitual use of the skill)
7	Using appropriate psychodynamic psychotherapy interventions per the patient’s state		C3: Applies the information A3: Values the learning P4: Mechanism (habitual use of the skill)
8	Managing the patient’s resistance in the session		C6: Creates new knowledge A3: Values the learning P4: Mechanism (habitual use of the skill)
9	Responding adequately to the changes in the patient’s emotional states		C6: Creates new knowledge A3: Values the learning P4: Mechanism (habitual use of the skill)
10	Managing the patient’s transference		C6: Creates new knowledge A3: Values the learning P4: Mechanism (habitual use of the skill)
11	Managing countertransference reactions		C6: Creates new knowledge A3: Values the learning P4: Mechanism (habitual use of the skill)
12	Summarizing and closing the session		C6: Creates new knowledge A3: Values the learning P4: Mechanism (habitual use of the skill)
Total Score			

*Bloom’s Taxonomy: C, cognitive; A, affective; P, psychomotor.

## 4 Discussion

This study described the psychometric properties of comprehensive assessment scales for the competency of psychodynamic psychotherapy for BPD among individuals who were trained as psychodynamic psychotherapists for BPD patients. The decision to design the scales in scoring rubrics was considered for the utility of rubrics in performance-based assessment. A scoring rubric objectively evaluates complex skills or behaviors by breaking them down into observable criteria (33, 34).

The CS-CAPC in psychodynamic psychotherapy for BPD was developed through the sequential activities of a Delphi survey, panel discussion, and validity testing. The scales in CS-CAPC in psychodynamic psychotherapy for BPD indicated satisfactory content validity, with S-CVI/Ave = 0.981 for the PF-CAS and S-CVI/Ave = 1.00 for the PC-CAS. The data collected during content validation indicated that all scale items were relevant for assessing the skills of psychodynamic formulation writing and conducting psychodynamic psychotherapy for BPD in real-life clinical settings. Most qualitative feedback was centered on creating a clear distinction between all the scores on the scale, allowing us to ensure an accurate grading of the assessed competency. Every revision of the scale was made based on participant feedback. One of the strengths of this study was the recruitment of a diverse sample of psychotherapy experts, psychiatrists experienced in psychotherapy, and teaching staff psychiatrists from nine psychiatry residency program institutions in Indonesia, allowing them to provide written feedback for the scale items during all activities. This helped us accumulate opinions and perspectives on the scales from the major stakeholders directly involved in learning situations across various institutions in Indonesia.

The CS-CAPC was designed to evaluate the competency of psychodynamic psychotherapy for BPD, as measured in the cognitive, affective, and psychomotor domains. This characteristic is among the novelties of our scale, as no previous scales for the competency of psychodynamic psychotherapy, let alone psychotherapy, defined the cognitive, affective, and psychomotor domains present in the task. The PF-CAS was used to evaluate the level of therapists’ competency in the cognitive and affective domains. The psychodynamic formulation is a tentative hypothesis that contains a succinct conceptualization of a patient’s clinical picture and guides the treatment plan (35). Our assignment of the cognitive and affective domains to the competency of writing a psychodynamic formulation was positively aligned with how creating a psychodynamic formulation requires the therapist’s willingness to delve into the patient’s internal world to discern central conflicts and themes in their lives (36). Mace and Binyon (37) stated that, in addition to gathering information from questioning, therapists might need to reflect on the feelings they experienced when interacting with a patient to infer the patient’s characteristic style of interpersonal relationships. These requirements correspond to the affective domain of learning and cognitive domain of understanding the theoretical framework of the psychoanalytic theories (20).

For the PC-CAS in psychodynamic psychotherapy for BPD, which assesses the cognitive, affective, and psychomotor domains, Ackerman and Hilsenroth (38) previously discussed how performing psychotherapy required a combination of cognitive, affective, and interpersonal skills. Several non-verbal elements are ubiquitous in psychotherapy, including eye contact, aligning body gestures per the patient’s emotional state, and voice and interruption behaviors (39, 40). This supports our assignment of the psychomotor domain to all items of the PC-CAS in psychodynamic psychotherapy for BPD.

The final version of the PF-CAS contained six items. The items on our scale reflect the structured components of a psychodynamic formulation as delineated in the literature. Sperry (41) defined three components of psychodynamic formulation: (1) a description of the patient’s clinical picture and stressors; (2) an etiological rationale for the factors contributing to the patient’s clinical picture; and (3) a formulation of treatment and prognosis based on the first two components. Similarly, a format proposed by Perry (42) and later updated by Summers (43) outlined the four essential components of a psychodynamic formulation: (1) summary of the patient’s problems; (2) description of the non-dynamic factors; (3) psychodynamic explanation of the patient’s central conflicts; and (4) prediction of the course of the therapy. The first item on our scale, “describing the patient’s condition and stressors,” summarizes the patient’s clinical picture and related stressors. The second item, “formulating the biological, intrapsychic, and sociocultural factors that influence the patient’s condition,” corresponds with describing the factors that contributed to the patient’s condition. The third item, “determining the suitable psychoanalytic theory to explain the patient’s condition,” embodies the formulation of the patient’s central conflicts using one or more psychodynamic theories. The fourth, fifth, and sixth items, “describing the potential resistance, transference, and countertransference that might occur in the therapy sessions,” “explaining the goals of the psychotherapy and the management plan for the potential resistance, transference, and countertransference that might occur in the therapy sessions,” and “explaining the patient’s prognosis in the course of the psychotherapy,” reflect the formulation of treatment and prognosis.

Our findings on the PF-CAS are consistent with those in previous studies that attempted to develop a method to assess written psychodynamic formulations. The case formulation content coding method (CFCCM) designed by Eells et al. (44) was used to evaluate the completeness and quality of the case formulation based on four content areas: (1) symptoms and problems; (2) the patient’s precipitating stressors; (3) predisposing life events; and (4) an explanation of the patient’s current difficulties by linking the three previous categories. The CFCCM rates the quality of the formulation as a whole and in the three dimensions of complexity, degree of analytic inference, and precision of language on a 5-point scale (44). Our rating scale contains a combination of the dimensions in the CFCCM’s rating scale, with the highest level of 3 representing the most comprehensive, analytical, and easily understood explanation of each component. Other models have focused on assessing the completeness of a patient’s psychodynamic conceptualization, such as Perry et al.’s (45) idiographic conflict formulation method and Curtis et al.’s (46) plan diagnosis method. The PF-CAS assesses the components of the patient’s psychodynamic conceptualization, similar to these existing methods, and it adds items that evaluate the hypothetical course of the therapy and treatment plan.

The PC-CAS in psychodynamic psychotherapy for BPD comprises 12 items: (1) beginning the session; (2) paying attention to the patient without judgment and prejudice; (3) conducting conversations that aim to build rapport with the patient; (4) maintaining good eye contact throughout the therapy session for most of the time; (5) aligning the gestures and posture per the patient’s state during the therapy session; (6) paraphrasing the patient’s statements to show that the therapist is empathetic and understanding toward the patient’s feelings; (7) using appropriate PP interventions per the patient’s state; (8) managing the patient’s resistance in the session; (9) responding adequately to the changes in the patient’s emotional states; (10) managing the patient’s transference; (11) managing countertransference reactions; and (12) summarizing and closing the session. Our findings agree with previous evaluation scales for competency in psychodynamic psychotherapy (20, 22). The rating scale developed by Winer and Mostert (20) evaluates the following items: establishing a therapeutic situation; facilitating the formation of a therapeutic alliance; recognizing the therapist’s own emotional reactions; experiencing the patient’s feelings while maintaining objectivity; communicating an empathic understanding in a way that enables the patient to feel understood; detecting multiple meanings in the patient’s communication; making interpretations; and formulating a psychodynamic explanation for the patient. The 29-item supervisor’s evaluation scale contains items that assess residents’ capacity to establish a working alliance of empathy, recognize and interpret transference, deal with resistance and countertransference, and close the session (22). The new item proposed in the PC-CAS in psychodynamic psychotherapy for BPD assesses a therapist’s ability to respond to a patient’s emotional changes. Maroda (47) and Kernberg (48) have stated that a therapist’s capacity to respond to a patient’s emotions plays a vital role in the psychodynamic psychotherapy of borderline patients, as emotional dysregulation causes these patients to exhibit frequent bouts of “affect storms.”

The PC-CAS in psychodynamic psychotherapy for BPD also emphasized the management of transference and countertransference in the psychodynamic psychotherapy of BPD. Transference interpretations may be viewed as “high-risk, high-gain” interventions in the psychotherapy of borderline patients, as these patients appear to be more vulnerable and easily overwhelmed by transference interpretations (9, 49). Transference interpretations in the psychodynamic psychotherapy of borderline patients must be performed in a timely and appropriate yet still evocative manner, to propel the process of psychotherapy (9, 20, 49). These features are consistent with the emphasis on timing and directed elaboration when managing transference in the PC-CAS in psychodynamic psychotherapy for BPD. Working with borderline patients is known to elicit a wide array of strong countertransference reactions within the therapists, including rage and hatred, guilt, inadequacy, anxiety, and parental feelings (9, 49). A meta-analysis by Hayes et al. (50) found that the management of countertransference was related to better therapeutic outcomes. However, the scope of this meta-analysis was not restricted to psychodynamic psychotherapy and borderline patients. The countertransference factors inventory (CFI) measures therapists’ countertransference management behavior by assessing five attributes related to countertransference management: self-insight, self-integration, anxiety management, empathy, and conceptualizing ability (51). The characteristics of self-insight, self-integration, anxiety management, and conceptualizing ability are reflected within the highest score of Item 11 on the PC-CAS in psychodynamic psychotherapy for BPD. Meanwhile, the empathy attribute is reflected in Item 6 on the PC-CAS in psychodynamic psychotherapy for BPD.

Our study had several strengths and limitations. The first strength is the robust data collection from a diverse sample of psychotherapy experts, psychiatrists experienced in psychotherapy, and teaching staff psychiatrists in Indonesia. The number of samples in the Delphi method, panel discussion, and content validity testing fulfilled the minimum number of participants for the respective activities (31, 52–54). Another strength of this study lies in the comprehensive steps taken in developing and validating the scales, including collecting qualitative feedback. The limitation of the study is the small number of experts in the panel discussion due to the limited number of psychotherapy consultants in Indonesia. The scales demonstrated potential utility in assessing the specific competency of psychodynamic psychotherapy for BPD and could be used in both learning and clinical situations. Future studies should investigate the application of these scales in education and clinical environments. The scales could be used for psychotherapy training or monitoring purposes and the results could be analyzed in comparison with other measures of psychotherapeutic competence, such as patient outcome.

## 5 Conclusion

The CS-CAPC in psychodynamic psychotherapy for BPD was designed to evaluate the cognitive, affective, and psychomotor domains of competency in psychodynamic psychotherapy for BPD, and it was found to have satisfactory content validity. The first scale in the CS-CAPC in psychodynamic psychotherapy for BPD, PF-CAS, could be used to assess the quality of written psychodynamic formulation. Meanwhile, the second scale, PC-CAS, assesses the practice of psychodynamic psychotherapy for BPD through direct or indirect observation via video recordings of the therapists’ psychotherapy sessions. The scale serves as a tool for a more objective and systematic assessment of the competency of psychodynamic psychotherapy for BPD, which could aid the didactic practice for psychodynamic psychotherapy for BPD among trainee therapists, such as psychiatry residents.

## Data Availability

The datasets presented in this article are not readily available because the participants did not agree to publicly share the data they provided for the study. Requests to access the datasets should be directed to ptrn1010@yahoo.com.
